# Contraceptive Use and Pregnancy Outcomes among Opioid Drug-Using Women: A Retrospective Cohort Study

**DOI:** 10.1371/journal.pone.0116231

**Published:** 2015-03-04

**Authors:** Charles S. Cornford, Helen J. Close, Roz Bray, Deborah Beere, James M. Mason

**Affiliations:** 1 Fulcrum Medical Practice, Acklam Road, Middlesbrough, England; 2 School of Medicine, Pharmacy and Health, Wolfson Research Institute, Durham University, Stockton-on-Tees, England; 3 Clinical Blood Sciences, Jenner Wing, St Georges Hospital, Blackshaw Road, Tooting, London,England; 4 Northern Region Gender Dysphoria Service, Benfield House, Walkergate Park, Benfield Road, Newcastle upon Tyne, England; UCL, UNITED KINGDOM

## Abstract

**Objective:**

The contraceptive needs of illicit opioid users differ from non-drug users but are poorly understood. The aim of this study was to describe contraceptive use and pregnancy outcomes in opioid-using women, and to examine their association with a range of risk factors.

**Method:**

This retrospective cohort study used UK general practice records, Treatment Outcomes Profile and National Drug Treatment Monitoring System data, and a nested data validation exercise. A cohort of 376 women aged 20–61 years were in active treatment for opioid addiction in October 2010 at two specialised primary care practices in North-East England. Outcomes were age-adjusted prevalence estimates for contraceptive use and pregnancy outcomes in users of illicit opioids. The association between lifestyle-related risk factors and contraception was explored.

**Results:**

Drug-using women made lower use of planned (non-condom) contraception (24% vs 50%, p<0.001), had more frequent pregnancy terminations (0.46 vs. 0.025, p = 0.004) and higher annual incidence of chlamydia (1.1% vs. 0.33%, p<0.001), when compared with age-matched population data. Specifically, there was low use of oral contraceptives (4% vs. 25%, p<0.001), IUCD (1% vs. 6%, p<0.001), and sterilisation (7% vs. 6%, p = 0.053), but higher rates of injectable contraceptives (6% vs. 3%, p = 0.003). A total of 64% of children aged <16 years born to this group did not live with their mother. No individual risk factor (such as sex-working) significantly explained the lower use or type of non-condom contraception.

**Conclusions:**

This is the first study to describe planned contraceptive use among drug-users, as well as the association with a range of risk factors and pregnancy outcomes. The low uptake of planned contraception, set against high rates of terminations and sexually transmitted disease demonstrates the urgent clinical need to improve contraceptive services, informed by qualitative work to explore the values and beliefs influencing low contraceptive uptake.

## Introduction

In England, women addicted to heroin or crack cocaine account for 4.05/1000 of the population (aged 15–64) [1]. Heroin use has devastating effects for both men and women, but women are subject to a whole range of additional challenges relating to sexual relations with men, contraception, pregnancy and child bearing [2–8]. Preconception and prenatal drug and alcohol use may directly cause poor maternal outcomes as well as adverse effects on the developing foetus, with life-long consequences [2]. Multiple sexual partners, sex work, chaotic life styles, and a high prevalence of sexually transmitted disease impact both on the type of contraception used and the type that would be most appropriate [4]. Women’s attitudes towards contraception may be affected by several factors, including amenorrhoea, fatalistic ideas and problematic relationships with men [5]. However, little is known about the choices drug-using women make about their contraception, or about subsequent pregnancy outcomes. Much evidence is drawn from undifferentiated populations in which drug use is commonplace.

Within the UK general population, in 2008/09, around 75% of women under 50 were using at least one method of contraception with the contraceptive pill and the male condom cited as most popular methods of contraception (25% each), followed by partner sterilisation (11%) and self-sterilisation (6%). Around 25% were not using contraception, of whom just over a half (13%) reported not being engaged in a heterosexual relationship [9]. These trends are reflected worldwide, apart from on the African continent in which contraception use is around 30% [10].

There is some knowledge about patterns of contraception use in drug using groups and in groups sharing similar features [11]. In a group of women in treatment for drug addiction in America, 76% of pregnancies were unplanned and 62% of the women having sex in the previous month had not used any contraceptive [12]. The use of non-condom contraception is surprisingly low in sex-workers: only 48% of parlour-based workers and 11% of street workers reported using contraception in addition to condoms [13]. A survey of contraceptive use in hepatitis C positive women in Australia found that only 34% used any form of contraception compared to 67% in a national survey [14]. Of those with hepatitis C and using contraception, 50% used barrier methods compared to 21% in the national survey. Incarcerated women have poor use of any contraception, and high rates of unplanned pregnancies, multiple partners and Sexually Transmitted Infections (STIs) [15]. A survey of intravenous drug users in New York showed 26% reported any contraceptive use compared to 48% use in women based on a national survey [16].

Although appropriate condom use is proven to reduce the risk of sexually transmitted infections, non-condom contraception may be more acceptable for women who have chaotic life styles or are intoxicated at the time of intercourse [17]. However, the uptake of non-condom contraception in this population remains unknown. Research into contraceptive use amongst opioid drug users is largely dated, does not make a clear distinction between condom and non-condom use, and assumes that drug users are a homogenous group in terms of their lifestyles, drug choices, behaviours and medical profiles. There is limited evidence supporting the need for a more personally tailored and heterogeneous approach taking into account use of illicit drugs, medication and the personal context of the drug user. Nevertheless, there is a particular need for effective contraception in this group. The effects of drug use in pregnancy are difficult to determine because of a wide range of confounding variables including poly-drug use. Cocaine and heroin use in pregnancy are associated with intra-uterine growth retardation; alcohol use, which is associated with illicit drug use, additionally has well-known teratogenic effects [2] [6]. Mothers using opiates are particularly concerned about neonatal withdrawal syndrome [7]. Successful pregnancies may, in some cases, lead to a further range of potential problems including pressures on other family members to take on the care of children, legal proceedings and the ‘bereavement’ of children being taken into care [3] [8] although the relationship between child protection issues and contraception remains complex and poorly described. For example, the permanent residence of children born to this population (a proxy for child protection proceedings) is poorly understood within both UK and non-UK contexts.

As well as the need for effective contraception, effective protection against STIs is often required. Intravenous drug use is associated with hepatitis C and HIV, and high levels of other sexually transferrable infections have been documented in incarcerated women [4] [11]. Multiple sexual partners and a high prevalence of sexually transmitted diseases were found in a study of drug treatment services in America over 20 years ago [12]. Drug use is common in sex workers, particularly in street-based workers in whom it is almost universal [18]. Within this group, use of crack cocaine makes condom use less likely [17].

A range of problems associated with contraception have been demonstrated in drug-using, or related, groups. Poor knowledge about contraception includes exaggerated worries about the dangers of contraceptive use and fatalistic views about the occurrence of pregnancy [17]. Amenorrhoea is induced by heroin (and to a lesser extent by methadone) through the hypothalamic-pituitary axis and is aggravated by loss of weight [5]. This may also impact on attitudes of women who may then believe their fertility is low [19]. Troubled childhood and adolescence, coercive relationships with men including sex in exchange for drugs and male dominated cultures are some of the difficulties faced by women using crack cocaine [8]. The high levels of mental health disorders in opiate users [20] may also impact on contraceptive use. Other difficulties include the need to trust despite experiencing fragile relationships, and the common occurrence of past and present violence, rape and sexual abuse [11]. Women in this group frequently experience grief from previous losses, poor self-esteem, feelings of shame, poor self-efficacy, poor social and negotiating skills, chaotic life styles, difficulty in keeping appointments and a general mistrust of health professionals who either may stigmatise drug users or misunderstand them [8][11][12].

Thus this is the first study to describe planned contraceptive use among a British cohort of opioid drug-users and its potential associations with a range of behavioural, relationship, physical and psychiatric risk factors. Additionally, this study presents a unique opportunity to describe pregnancy outcomes (including the residential destination of the child). This will inform future research into the complex, multi-factorial relationships between family planning and pregnancy outcomes.

## Method

The primary aims of the study were descriptive: to provide crude, and age adjusted prevalence estimates for contraceptive use and pregnancy outcomes in women currently receiving opioid-substitution treatment (OST) for addiction to illicit opioids (injecting and non-injecting) in a specialist community care setting. The secondary aim was to explore the association between current heroin use and use of planned (non-condom) contraceptives, adjusting for a range of behavioural, social, physical and psychiatric risk factors.

### Sample and setting

A convenience sampling frame was adopted in which contraceptive and pregnancy histories were retrieved for all 376 female patients receiving OST (methadone or buprenorphine) and who were registered for general medical services (GMS) at two Medical Practices in Middlesbrough and Stockton-on-Tees, which serve a region of relative deprivation in the north of England. Data extraction occurred in June 2011. The practices both provide specialist treatment services for opioid addiction to patients from the surrounding area. These practices had a total of 1,332 patients at the time of the survey who either had drug addiction problems requiring medical treatment or were children of such patients. The practices are otherwise ‘standard’ general practices, offering the usual range of primary care services. The specialist Middlesbrough practice provided OST for all patients receiving treatment for opiates in the surrounding area. These patients could be registered for general medical services (GMS) at the specialist practice or at other practices in the Middlesbrough area: this study only includes patients registered at the specialist practice. Previous research has demonstrated no significant differences in terms of age, gender or ethnicity according to where OST patients receive GMS [21]. The percentage of female patients receiving OST was 30% and mean age was 33: both similar to national figures [1].

### Measures

The Treatment Outcomes Profile (TOP) measure is a validated self-report tool, routinely completed in UK drug treatment settings, assessing change and progress in key areas of the lives of people in treatment for drug addiction. TOP includes: frequency, mode, and type of illicit drug use; social functioning (housing, employment, and education status); subjective health and psychological status; criminal activity; and, activities and behaviours that have occurred in the preceding 4 weeks [22]. A TOP measure was completed for each patient every 3 months, with the most recent being used in the analysis. Use of illicit drugs within the preceding 4 weeks was used to define ‘current’ drug use.

Data from the National Drug Treatment Monitoring System form [23] was also available for all patients in treatment. This is completed by all drug treatment agencies in England in order to monitor performance against local and national targets; these included information such as ethnicity, hepatitis B vaccination status, hepatitis C testing status, illicit drugs used, and sex-worker status. Data were extracted from the practice records using a search, including: contraceptive use, pregnancies, terminations, miscarriages, still and live births, family composition, sexually transmitted diseases, cervical testing and sex work.

Contraceptive use was defined as the method recorded in GP prescription records, and non-barrier contraception was defined as any record of any non-barrier method. Contraception was classified as ‘current’ or ‘past’ according to whether use would still have been active. For example if a six month prescription for the combined contraceptive pill was recorded less than six months before the time of the note search, this would be noted as ‘current’; if issued more than six months ago it would be recorded as ‘previous’.

### Validation

Assessing lifestyle and behavioural factors such as the level of abuse experienced by female opiate users is problematic since it depends upon self-report and the context in which information is sought. These factors are of particular interest because of their potential role in contraceptive choices. However, recording within the TOP data set was found to be too incomplete to provide a reliable assessment of such factors. Thus, a validation exercise was conducted at one of the two practices (including 176 patients), assessing previous involvement in sex work, sexual abuse, and domestic violence. A total of 10 keyworkers (health care worker responsible for the drug addiction treatment of individual patients) were interviewed and asked a series of questions with binary (yes/no) answers about each individual patient within the current cohort registered at their practice. Two meetings were arranged with the keyworkers in a group setting. They were asked to provide the information for each patient in turn. Each keyworker had worked with an individual patient for a length of time varying from weeks to many years. Some patients had received care from more than one keyworker at different periods, so the knowledge gained was a collective response to the questions. Three of the keyworkers were not at one or both of the meetings, and so they were asked on an individual basis to provide information for their patients.

### Analysis

A search was made of the GP records of all eligible female patients over the age of 18 currently in treatment for opiate addiction. Data capture was piloted by reviewing the notes of 10 eligible patients to identify data recording issues. Subsequently, data from sources were combined and anonymised before analysis; no individual patient characteristics were identifiable within the analyses. Analysis was conducted using SPSS (version 21). When comparing estimated proportions with population values, 2-sided single proportion tests were conducted against the null population value and exact confidence intervals (CIs) were estimated. When comparing cohort event rates with population values, indirect standardisation was employed, where the event rate in an age-matched population cohort is estimated and the subsequent standardised event ratio (observed/expected) is evaluated as the test statistic: = (O-E)^2^/E~*X* ^2^[1]. Simple univariable modelling included the full range of potential explanatory variables. Stepwise addition iterating to the best model did not prove necessary. All estimates of test probabilities are reported for the purpose of hypothesis generation with a 5% threshold for significance and thus without post-hoc correction. The prospective study power calculation estimated that approximately 30% of 300 adult female patients would be current users of contraception, providing a crude prevalence estimate with precision: 95%CI = 25% to 35%.

### Ethics

This study was approved (by Chair’s action) by South Tees NHS Research Ethics committee. Individual consent was not required, and patient data were anonymised and de-identified prior to analysis.

## Results

### Demographic characteristics

Data were available for 374 female patients who were receiving treatment for illicit opioid addiction; the mean age was 33 years (range 20–61 years) and 353 (94%) were recorded as white British. All patients had previously used heroin. At initial entry into treatment, the cohort of women had been recorded as using (non-exclusively) heroin in 337 (90%) (smoked by 168 (45%), injected by 150 (40%)), crack cocaine in 101 (27%), methadone in 30 (8%), benzodiazapines in 15 (4%) and cannabis in 15 (4%); other drugs were used less frequently. The average age of beginning drug use was 21 years (range 12 to 40). Hepatitis C testing had been conducted for 269 (72%) of patients and found to be present in 82 (22%) of patients. Workforce participation was limited with 19 (5%) of the sample in regular employment, 247 (66%) unemployed, long term sick or disabled, and 108 (29%) undisclosed.

Rates of completion of cervical smear testing were high in this group, with a record of 307 (82%) uptake within the previous three years, comparing favourably to the 79% uptake nationally for women aged 25–64 [24]. Chlamydia had been diagnosed in 33 (8.9%) of subjects tested; based on the previous three years the annual incidence was 1.1% (95% CI: 0.5% to 1.9%). Nationally, the population annual incidence of chlamydia is 0.33% [25], one third of the rate in this cohort (1.1% vs 0.33%, p<0.001). Numbers were too small to reliably interpret other disease rates against population figures.

### Contraceptive use

Data were available for 376 female participants receiving OST. Current overall contraceptive use was low at 113 (30%) when compared to a national age-standardised figure of 75%, although this figure includes recorded condom use (Table 1). However, the use of non-condom contraception was similarly low (90 (24%) compared to 50% in the population, p<0.001). More specifically, there was low use of oral contraceptives (15 (4%) compared to 25%, p<0.001), IUCD (4 (1%) compared to 6%, p<0.001), similar rates of sterilisation (26 (7%) compared to 6%, p<0.053) but higher rates of injectable contraceptives (23 (6%) compared to 3%, p = 0.003). (All comparisons based on age-adjusted national data [26]).

**Table 1:** Current and previous contraception use.

	Age Category									
	20–29		30–39		40–49		50+		Total	
	N = 137		N = 189		N = 41		N = 7		N = 374	
Current Contraceptive Use	39	29%	58	31%	12	29%	1	14%	110	29%
Condoms (male)	12	9%	14	7%	0	0%	0	0%	26	7%
Condoms (female)	0	0%	0	0%	0	0%	0	0%	0	0%
Non-condom contraception	31	23%	47	25%	11	27%	1	14%	90	24%
Diaphragm/cap	0	0%	0	0%	0	0%	0	0%	0	0%
Combined pill/patch/vaginal ring	8	6%	6	3%	1	2%	0	0%	15	4%
POP pill	2	2%	5	3%	2	5%	0	0%	9	2%
Injectable	12	9%	7	4%	2	5%	0	0%	21	6%
Implant	7	5%	8	4%	0	0%	0	0%	15	4%
IUCD	0	0%	2	1%	0	0%	0	0%	2	1%
IUS	0	0%	3	2%	0	0%	0	0%	3	1%
Sterilisation	3	2%	15	8%	7	17%	1	14%	26	7%
Vasectomy (partner)	1	1%	0	0%	1	2%	0	0%	2	1%
Other	1	1%	2	1%	0	0%	0	0%	3	1%
Previous Contraception Use	100	73%	145	77%	22	54%	1	14%	268	72%
Condoms (male)	50	37%	70	37%	6	15%	0	0%	126	34%
Condoms (female)	2	2%	2	1%	0	0%	0	0%	4	1%
Non-condom contraception	86	63%	129	68%	23	56%	1	14%	239	64%
Diaphragm/cap	0	0%	0	0%	0	0%	0	0%	0	0%
Combined pill/patch/vaginal ring	46	34%	77	41%	14	34%	0	0%	137	36%
POP pill	9	7%	17	9%	4	10%	0	0%	30	8%
Injectable	51	37%	81	43%	10	24%	0	0%	142	38%
Implant	12	9%	14	7%	0	0%	0	0%	26	7%
IUCD	4	3%	6	3%	4	10%	1	14%	15	4%
IUS	1	1%	4	2%	0	0%	0	0%	5	1%
Other	1	1%	1	1%	0	0%	0	0%	2	1%
Emergency contraception	23	17%	39	21%	1	2%	0	0%	63	17%
Number of occasions^1^	2.2		2.2		1.0		0.0		2.2	

^1^Mean number of occurrences within each age category

### Pregnancies and family composition

Most subjects (323, 86%) had become pregnant at least once, with an average of 3.31 pregnancies and 2.43 live births in those becoming pregnant (Table 2). Thus live births were not dissimilar to the population norm. Using population data from England and Wales in 2010 for mothers going to term, the average number of children expected for an age-matched cohort was 1.97 [27], providing a Standardised Birth Ratio of 1.24, p = 0.74. However, the termination rate was 0.46 per mother within the total cohort, far exceeding an age-matched population cohort of 0.025 per mother (p = 0.004).

**Table 2:** History of Pregnancy.

	Age Category									
	20–29		30–39		40–49		50+		Total	
	N = 137		N = 189		N = 41		N = 7		N = 374	
Pregnancy	110	80%	167	88%	39	95%	6	86%	322	86%
Number of pregnancies^a^	2	2	3	2	3	3	4	5	3	3
Live births^b^	91	82%	159	95%	39	100%	5	83%	294	91%
Number of live births ^a^	1	2	2	2	2	2	3	4	2	2
Terminations ^b^	33	30%	62	37%	15	39%	2	33%	111	35%
Number of terminations ^a^	0	0	0	1	0	1	0	1	0	1
Ectopic pregnancies ^b^	3	3%	3	2%	1	3%	0	0%	8	3%
Number of ectopic pregnancies ^a^	0	0	0	1	0	0	-	-	0	0
Miscarriages ^b^	26	25%	53	32%	10	26%	4	67%	92	29%
Number of miscarriages ^a^	0	0	0	1	0	1	1	1	0	1
Still births ^b^	2	2%	3	2%	2	5%	0	0%	7	2%
Problems becoming pregnant	7	7%	16	10%	3	7%	1	14%	27	10%
Referral for secondary care investigation ^c^	4	31%	10	63%	2	67%	1	100%	17	53%
Referral for reversal of Sterilisation ^c^	1	8%	1	5%	0	0	0	0%	2	6%

^a^ Median number (interquartile range) of events in those with at least one event

^b^ Proportion of mothers experiencing an event who have become pregnant at least once

^c^ Proportion of mothers referred because of problems becoming pregnant

An analysis of family composition by age shows unsurprisingly that family size grows with age but that the majority of children under the age of 16 do not live with their maternal mother (Table 3). Within the cohort, of 556 children aged under 16, 203 (36%) lived with their mother, 217 (39%) lived instead with their father or other family member and 113 (20%) had been adopted or were in foster care.

**Table 3:** Family composition according to mean (median/Interquartile range) number of children per age band.

	Age Category (Mean/median/IQR)														
	20–29			30–39			40–49			50+			Total		
	N = 92			N = 159			N = 39			N = 5			N = 295		
	$\bar{x}$	$\tilde{x}$	IQR	$\bar{x}$	$\tilde{x}$	IQR	$\bar{x}$	$\tilde{x}$	IQR	$\bar{x}$	$\tilde{x}$	IQR	$\bar{x}$	$\tilde{x}$	IQR
Progeny aged 16+	0.02	0	0–0	0.38	0	0–1	2.03	2	1–3	2.80	2	1–4	0.52	0	0–1
Children (aged <16) living with mother	0.66	0	0–1	0.74	0	0–1	0.51	0	0–1	0.20	0	0–0	0.68	0	0–1
Children not living with mother but living with father	0.13	0	0–0	0.13	0	0–0	0.00	0	0–1	0.00	0	0–0	0.11	0	0–1
Children not living with mother but living with other family member	0.75	0	0–1	0.68	0	0–1	0.05	0	0–0	0.00	0	0–0	0.61	0	0–0
Children in foster care	0.22	0	0–0	0.36	0	0–0	0.18	0	0–0	0.00	0	0–0	0.28	0	0–0
Children adopted	0.10	0	0–0	0.12	0	0–0	0.03	0	0–0	0.00	0	0–0	0.10	0	0–0
Children under 16 not included in above	0.03	0	0–0	0.13	0	0–0	0.03	0	0–0	0.20	0	0–0	0.09	0	0–0

### Historical level of abuse

General practice note review reported previous involvement in sex work (44, 12%), sexual abuse (61, 16%) and domestic violence (113, 30%). A validation enquiry amongst keyworkers of client history resulted in far higher estimates (Table 4).

**Table 4:** Rates of abuse experienced in the past year and ever.

	Sex work		Sex abuse		Domestic violence	
	Notes	Keyworker^1^	Notes	Keyworker^1^	Notes	Keyworker^1^
Ever	44 (11.7%)	52 (29.5%)	61 (16.3%)	53 (30.1%)	113 (30.1%)	72 (40.8%)
Last year	5 (1.3%)	23 (13.1%)	7 (1.9%)	14 (8.0%)	17 (4.4%)	36 (20.2%)

^1^ Taken from validation exercise at practice A (n = 176 patients)

### Associations with planned contraceptive use

The influence of a range of patient risk factors upon planned contraceptive use was explored (Table 5). Only history of sexual abuse was statistically significantly associated with greater planned contraception (p = 0.03). A similar but non-statistically significant positive trend is apparent for recent sexual abuse and domestic violence, while there was no statistically significant association between lower planned contraceptive use with current opiate use (for any use or heavy use) and sex-work.

The range of patient risk factors was further explored within sub-groups of different types of non-condom contraception (Table 5). Similar patterns are observed although smaller numbers affect statistical significance. The use of IUCDs was greater with heavy cannabis use (p = 0.03) and combined pill use was more common in alcohol users although confidence intervals were wide (95% CI 1.5 to 427.4, and 1.09 to 10.7 respectively).

**Table 5:** Determinants of planned contraceptive use by type: univariable (simple) regression model findings*.

	Non-condom contraceptive use (ALL)				Pill				IUCD				Injectables				Sterilisation			
	N	OR	95%CI	p	N	OR	95%CI	p	N	OR	95%CI	P	N	OR	95%CI	p	N	OR	95%CI	p
**Age**	90	1.01	(0.97 to 1.04)	0.77	15	0.90	(0.81 to 1.00)	0.77	2	1.03	(0.84 to 1.25)	0.79	21	0.93	(0.86 to 1.02)	0.11	26	1.12	(1.06 to 1.18)	0.001
**Alcohol Use^1^**	78	1.09	(0.64 to 1.87)	0.75	13	3.41	(1.09 to 10.7)	0.04	2	2.03	(0.13 to 32.7)	0.62	21	1.26	(0.51 to 3.14)	0.62	23	0.88	(0.35 to 2.19)	0.78
**Heavy Alcohol Use^1^^,^^2^**	78	0.75	(0.27 to 2.06)	0.58	13	2.21	(0.46 to 10.6)	0.32	2	-			21	1.25	(0.27 to 5.67)	0.77	23	-		
**Opiate Use^1^**	81	0.61	(0.35 to 1.07)	0.084	14	0.38	(0.08 to 1.77)	0.22	2	1.57	(0.11 to 28.2)	0.69	21	0.94	(0.36 to 2.41)	0.89	25	0.41	(0.15 to 1.13)	0.08
**Heavy Opiate Use^2^**	78	0.57	(0.26 to 1.28)	0.17	14	0.98	(0.21 to 4.50)	0.98	2	5.95	(0.37 to 96.9)	0.21	20	0.60	(0.14 to 2.66)	0.50	25	0.49	(0.11 to 2.14)	0.34
**Crack cocaine use^1^**	82	0.98	(0.43 to 2.27)	0.97	14	0.79	(0.10 to 6.3)	0.83	2	-			21	0.92	(0.20 to 4.12)	0.91	25	1.28	(0.36 to 4.5)	0.71
**Heavy Crack cocaine use^2^**	79	0.70	(0.15 to 3.32)	0.66	12	-			2	-			21	1.57	(0.19 to 12.8)	0.68	24	-		
**Cannabis use^1^**	82	1.08	(0.50 to 2.31)	0.85	14	1.29	(0.28 to 5.96)	0.75	2	7.8	(0.48 to 127)	0.15	21	0.79	(0.18 to 3.54)	0.76	25	1.04	(0.30 to 3.66)	0.95
**Heavy Cannabis use^2^**	82	1.29	(0.39 to 4.24)	0.67	14	-			2	25.3	(1.5 to 427.4)	0.03	21	2.73	(0.57 to 13.07)	0.21	25	-		
**Intravenous drug use^1^**	82	0.47	(0.19 to 1.14)	0.096	14	1.14	(0.25 to 5.29)	0.86	2	-			21	-			25	0.57	(0.13 to 2.52)	0.46
**Sex worker^3^^,^^4^**	40	0.47	(0.13 to 1.66)	0.24	8	-			2	-			5	-			17	0.39	(0.05 to 3.06)	0.37
**Sex worker^1^^,^^4^**	40	0.78	(0.35 to 1.75)	0.55	8	-			2	-			5	0.59	(0.06 to 5.41)	0.64	16	0.78	(0.24 to 2.54)	0.68
**Sexual abuse^3^^,^^4^**	41	1.94	(0.61 to 6.17)	0.26	8	1.7	(0.19 to 14.9)	0.63	0	-			5	-			17	1.63	(0.33 to 7.99)	0.55
**Sexual abuse^1^^,^^4^**	41	2.24	(1.08 to 4.63)	0.030	8	2.43	(0.59 to 10.1)	0.22	0	-			5	0.57	(0.06 to 5.24)	0.62	17	1.72	(0.62 to 4.79)	0.30
**Domestic violence^3^^,^^4^**	40	1.72	(0.76 to 3.92)	0.20	8	4.32	(1.02 to 18.2)	0.05	0	-			5	-			17	1.75	(0.57 to 5.35)	0.33
**Domestic violence^1^^,^^4^**	40	1.63	(0.80 to 3.31)	0.18	8	2.53	(0.58 to 10.9)	0.22	0	-			5	0.97	(0.16 to 5.94)	0.97	17	2.25	(0.81 to 6.22)	0.12
**Physical Health^5^**	82	0.99	(0.93 to 1.05)	0.72	14	1.06	(0.93 to 1.21)	0.41	2	1.04	(0.74 to 1.46)	0.81	21	0.96	(0.87 to 1.07)	0.47	25	1.03	(0.93 to 1.13)	0.62
**Psychiatric Health^5^**	82	0.97	(0.92 to 1.03)	0.32	14	0.99	(0.88 to 1.12)	0.91	2	1.02	(0.74 to 1.40)	0.92	21	0.98	(0.89 to 1.08)	0.69	25	0.97	(0.88 to 1.06)	0.48
**Global Health^5^**	82	1.00	(0.94 to 1.05)	0.89	14	1.11	(0.97 to 1.3)	0.11	2	0.99	(0.73 to 1.35)	0.95	21	0.96	(0.87 to 1.05)	0.34	25	0.99	(0.91 to 1.09)	0.91

^1^ Ever

^2^ More than 1 day in 2

^3^ In the last year

^4^ Keyworker assessed (N = 176 patients)

^5^ Assessed using TOPS data

*Model constant not shown: Cocaine, Amphetamine and other drug use not reported because of small numbers of users

- Numbers too small to analyse

## Discussion

### Summary

This is the first cohort study to describe and evaluate the use of non-condom contraception in illicit drug-using women in the UK. When compared to the general population, non-condom contraceptive use was low in this group overall, with low use of oral contraceptives, IUCD, similar rates of sterilisation, but high rates of injectable contraceptives. Within group and sub-group analysis of age, alcohol use, current illicit opiate use, other illicit drug use, or reported health status did not explain these patterns of contraceptive use, although it is possible that the current cohort is underpowered to find modest but important influences. Rates of chlamydia infection were high, as were reported levels of domestic violence, past sexual abuse and sex work. Our findings suggest a significant under-recording of domestic violence, sexual abuse, and sex worker status. Findings also showed high levels of ectopic pregnancies, high rates of terminations, and two-thirds of children under 16 unable to live with their mothers. Thus, there is an urgent need to improve contraceptive services for this vulnerable group. Given the lack of observable risk factors or evidence for observed behaviours qualitative research may be appropriate to help understand contraceptive uptake and how contraceptive services might be improved in this group. The higher rates of injectable contraceptives in this group suggests a possible route for further enquiry, as does the high rate of cervical smears suggesting unexpectedly high levels of engagement: such clinical encounters may offer a particular opportunity for contraceptive service development. It is possible that the high level of one-to-one long-term support offered at the study treatment facilities gave women the opportunity to build a level of trust and positive engagement with service providers that could be built upon for other health-related interventions. Improving contraceptive uptake would benefit patients and have broader societal benefits.

### Strengths and limitations

Secondary use was made of medical records not designed for research purposes within retrospective analysis. The most obvious knowledge gap concerns the use of condoms. Practice record recording of non-condom contraceptive use within the British GP setting is reliable since provision is by prescription or would be recorded if provided by community contraceptive clinics. Recording of condom-based contraception is less reliable and thus the low current overall contraceptive use of 30% (compared to national figures of 77%) may be underestimated. Recording of sexual abuse, domestic violence and sex working status is likely to be variable and we have modelled formal and informal records to help explore these important factors. The Treatment Outcomes Profile (TOP) [22] measure was used as a proxy for mental ill-health and as such it has some important limitations. It measures psychological health on a 20 point scale and thus does not systematically capture psychiatric morbidity which may have been missed within the current study design. Similarly, data from the National Drug Treatment Monitoring System [23] was collected for monitoring purposes rather than primary research and thus the information collected was limited compared to the richer data provided during key worker interviews. For example, assessing the level of abuse experienced by female opiate users depends upon self-report and the context in which information is sought: recording within the TOP data set and GP note recording was too incomplete for research use. Thus, this is the first study to provide a validation of practice records for domestic violence, sexual abuse (recent and historical), and sex worker status via interviews with keyworkers. Although this type of reporting may be subject to recall bias, the level of dissonance between the records and keyworker knowledge suggests limitations in GP recording that warrant further investigation. It is possible that interviews with drug-using women themselves may have revealed further levels of information not available in routine datasets: we plan to conduct interviews in future research. Nonetheless the relatively large cohort of opioid users permits factors affecting contraceptive use to be meaningfully explored.

Failure to find any factors significantly explaining differences in planned contraceptive use was surprising. It would be expected that patients with more ‘chaotic’ lifestyles, such as heavy current alcohol use, current use of illicit opiates or domestic violence, might be less able to take advantage of planned contraception. Presumably latent group differences between opioid using women and non-opioid using women provide the explanation for this finding, but we are unable in the current study to account for what those differences might be. A hypothesis generating approach permits multiple testing to explore associations and relationships. However findings require replicating in further research to differentiate associations and artefact findings. Furthermore we accept that all epidemiological enquiry is vulnerable to confounding by omitted variables (unmeasured influences).

### Comparison with existing literature

Previous studies in groups including women treated for opioid addiction, such as sex workers, incarcerated women and women treated for hepatitis C infection, have previously suggested a low level of non-condom contraception although the true extent of this was unknown [13][14] [15]. This is the first study to our knowledge to describe and evaluate the low levels of non-condom contraceptive use in women in treatment for opioid addiction in the UK, a country where such contraception is widely and freely available, and whose uptake is not inhibited by negative social norms. This study showed a higher than usual current use of injectable contraceptive use. A previous survey of postnatal contraception in opioid using women suggested that this group failed to continue with injectable contraception and that this might be an inappropriate contraceptive [28]. However, there may be differences between opioid-using women in general and postnatal opioid-using women, warranting further exploration.

High rates of sexually transmitted infections have been documented in related groups, such as incarcerated women and drug treatment services in America, thus a high rate of chlamydia infections was not unexpected. Similarly, high rates of unplanned pregnancies were reported over 20 years ago, but this is the first study to our knowledge to show high rates of pregnancies specifically in the drug using population. Accurate population data are not available for pregnancy related outcomes to permit further comparison although rates of ectopic pregnancies appear high compared with prevalence estimates of around 1% based on acute hospital admission data [29]. Despite perceptions about lack of engagement with formal services in this group, there was a high cervical smear rate, mirroring that for women serving long-term prison sentences [30]. This suggests that despite challenging, chaotic circumstances, women can engage with personal and sexual health services, offering a possibility for future service development.

This study is the first known study to describe the prevalence of children living apart from their mothers in this population. Residence is an important but imperfect proxy for child protection proceedings and it is important to highlight that the study was not designed to examine the complex and multi-factorial association between contraceptive use and parenting breakdown or child protection issues. Developing an understanding of place of residence simply provides context within which to inform future research into the development of appropriate patient-centred services for this population.

### Implications for Practice

There is an urgent clinical need to improve contraceptive services amongst drug users, with the aim of reducing termination rates, ‘unseen’ consequences of a failure to plan such as ectopic pregnancies, and the maternal and foetal development disorders associated with drugs and alcohol during conception and pregnancy. In this study, the lack of evidence for within-group associations for contraceptive use demonstrates that factors such as current drug or alcohol use, perceived health status, sexual abuse or domestic violence should not be a barrier to uptake of such services within the group of opiate using women. The high uptake of cervical smear testing also demonstrates that it is possible to deliver high quality medical services within this group and presents a possible opportunity in clinical practice. The high incidence of chlamydia infection demonstrates the need for sexually transmitted disease services for this group. Further research is needed to explore the factors that influence contraceptive use in opiate using women, with the aim of developing more appropriate services to reduce the burden of unplanned pregnancies and STDs.
